# Movement-Based Interaction Applied to Physical Rehabilitation Therapies

**DOI:** 10.2196/jmir.3154

**Published:** 2014-12-09

**Authors:** Juan Enrique Garrido Navarro, Victor Manuel Ruiz Penichet, María Dolores Lozano Pérez

**Affiliations:** ^1^Computer Science Research InstituteUniversity of Castilla-La ManchaAlbaceteSpain; ^2^Department of Computer SystemsUniversity of Castilla-La ManchaAlbaceteSpain

**Keywords:** exercise movement techniques, human–computer interaction, interaction devices, movement-based interaction, rehabilitation therapies

## Abstract

**Background:**

Health care environments are continuously improving conditions, especially regarding the use of current technology. In the field of rehabilitation, the use of video games and related technology has helped to develop new rehabilitation procedures. Patients are able to work on their disabilities through new processes that are more motivating and entertaining. However, these patients are required to leave their home environment to complete their rehabilitation programs.

**Objective:**

The focus of our research interests is on finding a solution to eliminate the need for patients to interrupt their daily routines to attend rehabilitation therapy. We have developed an innovative system that allows patients with a balance disorder to perform a specific rehabilitation exercise at home. Additionally, the system features an assistive tool to complement the work of physiotherapists. Medical staff are thus provided with a system that avoids the need for them to be present during the exercise in specific cases in which patients are under suitable supervision.

**Methods:**

A movement-based interaction device was used to achieve a reliable system for monitoring rehabilitation exercises performed at home. The system accurately utilizes parameters previously defined by the specialist for correct performance of the exercise. Accordingly, the system gives instructions and corrects the patient’s actions. The data generated during the session are collected for assessment by the specialist to adapt the difficulty of the exercise to the patient’s progress.

**Results:**

The evaluation of the system was conducted by two experts in balance disorder rehabilitation. They were required to verify the effectiveness of the system, and they also facilitated the simulation of real patient behavior. They used the system freely for a period of time and provided interesting and optimistic feedback. First, they evaluated the system as a tool for real-life rehabilitation therapy. Second, their interaction with the system allowed us to obtain important feedback needed to improve the system.

**Conclusions:**

The system improves the rehabilitation conditions of people with balance disorder. The main contribution comes from the fact that it allows patients to carry out the rehabilitation process at home under the supervision of physiotherapists. As a result, patients avoid having to attend medical centers. Additionally, medical staff have access to an assistant, which means their presence is not required in many exercises that involve constant repetition.

##  Introduction

### Background

Doctors, physiotherapists, and health care centers are increasingly incorporating video games and, more specifically, new technologies and methods into their rehabilitation therapies [1-3]. The result is a process that allows patients to recover impaired functions in an entertaining, useful, and effective way. Neurorehabilitation [4] is one of the areas in which this new type of therapy has been used in recent years. In particular, this new rehabilitation procedure has been applied with people with brain damage caused by cardiovascular accident, cerebral infarction, and neurodegenerative diseases such as Parkinson disease, multiple sclerosis, and Alzheimer disease. Although this therapy is currently regarded as unconventional, it has proved highly beneficial when applied in rehabilitation treatment [1,2]. Specifically, the results of a project conducted with patients with multiple sclerosis at Rey Juan Carlos University [5] revealed significant improvement when exercises based on video games were used. Thanks to this new therapy, many patients have been able to fully resume their activities of daily living, such as walking, jumping, or keeping their balance when standing up, that had previously caused them great difficulties.

The use of movement-based interaction is one of the most popular elements of video games that can be applied in health care settings. Specifically, this kind of interaction allows the creation of systems that offer patients the possibility of completing the rehabilitation process at home by following the exercises prescribed by qualified medical staff.

In this paper, we present a system called “BDRehab,” which takes into account how movement-based interaction can improve rehabilitation processes. Specifically, the system focuses on the rehabilitation of patients with neurodegenerative diseases or some form of brain damage (eg, traumatic brain injury, dementia, cerebral palsy). This type of patient is unable to do many of these exercises, such as getting up or sitting on a chair, walking in a straight line, picking up or moving objects. The system focuses on helping patients in the recovery of balance function. These actions, which are easy for people in good health, are complex for people with neurodegenerative and brain diseases. Therefore, rehabilitation and training in these cases represent an essential and important improvement milestone.

The capacity to detect movement enables patient movement to be tracked and analyzed. The results of analysis provide enough information to perform corrective actions. The correction can be achieved by comparing how each rehabilitation exercise is actually carried out with how it should best be done. Accordingly, if the result is correct, then the system communicates this to the patient; if not, the system creates specific advice and clarification in animated form to help the patient complete the rehabilitation process in the correct way. Regarding data collection, the information concerning patient progress in each exercise facilitates the creation of a useful statistical study for medical staff. The aim of this additional functionality is to report on the current state of each patient’s rehabilitation process.

The BDRehab system uses Kinect for Windows [6] to work with movement-based interaction. This device is a major development, as it provides users with the capacity to interact with systems through common and natural gestures. The medical world has found Kinect to be a useful tool with which to experiment to reach solutions and make improvements. A wide range of issues have been treated, from haptic problems [7] to rehabilitation in chronic diseases [8]. The system is generalizable to similar movement-based interaction devices, such as Asus Xtion Pro Live [9] and SoftKinectDepthSense [10].

The rest of the paper is organized as follows. Outstanding related studies are described and compared in the next subsection. Section 2 describes the system developed for rehabilitation at home, as well as its functionality and interface. The outcomes of the evaluation are described in section 3. Section 4 presents conclusions and final remarks.

### Related Studies

The emergence of devices offering movement-based interaction in casual environments such as video games has created great interest in their application in medical settings. Specifically, rehabilitation processes incorporating physical exercise comprise one of the main areas in which the research community has found a suitable environment in which to apply these techniques.

**Figure 1:**
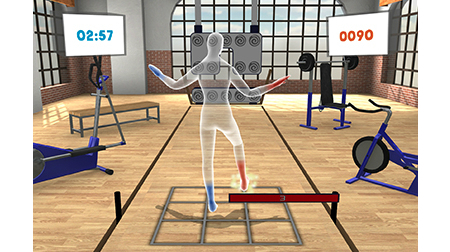
Example of exercises done in Virtual Rehab [11].

The first related study is outstanding. It describes a system, VirtualRehab [11], which involves physical rehabilitation using the movement-based interaction provided by Kinect. The system allows the monitoring and tracking of patients from any location. The main objective of VirtualRehab is to offer patients an enjoyable way of completing complex rehabilitation processes at home. To this end, the equipment required comprises a personal computer, Kinect for Windows software, and a screen. Additionally, VirtualRehab contains a manager that enables medical staff to plan, monitor, and review each patient’s progress. The system focuses on specific pathologies: acquired brain damage, neuromuscular diseases, neurodegenerative diseases, and mobility in older adults. To aid in the treatment of these pathologies, VirtualRehab places patients in a virtual world in which they can work with 9 games to perform specific movements to improve impairments (Figure 1). These games make it possible to work with the affected parts of the body and physical symptoms, particularly in patients with loss of motor ability, movement and posture disorders, and balance and coordination difficulties.

WiiHab is the second system we analyzed. In this case, the technology used is once again based on video games, but uses Nintendo technology through the Wii console. Specifically, WiiHab utilizes the potential of existing Wii games to encourage physical rehabilitation. Many studies [12-14] provide information on positive results with the use of WiiHab. Initially, WiiHab was focused on use of the basic controllers, named “Wii Remote,” to interact with the games. The main games used in physical rehabilitation were those related to sports: tennis, baseball, boxing, golf, and bowling. However, the evolution of the Nintendo console provides the possibility of interaction with new devices, such as the Wii balance board, with which the opportunities of dynamic activities (eg, aerobics or yoga) increase by the use of the feet as an element of interaction.

SeeMe [15] is another major study on rehabilitation, but in this case it corresponds to the assessment and treatment of unilateral spatial neglect. This system represents another method for rehabilitation that creates a virtual reality environment without the requirement of head-mounted displays or specialized equipment. SeeMe is a projected video capture of a virtual story in which patients are “embedded” through their own image. A representation of the patient is generated by capturing the individual through a camera while performing the activities. Specific algorithms are employed for movement and position recognition and analysis. The participants should stand or sit in a specific area while viewing a monitor on which their virtual representation is shown during the exercises inside the virtual world. For example, patients can be embedded in a game in which they are touching virtual exercise balls. Therefore, the participants can see themselves in a virtual story in which they use trunk and limb movements. Additionally, the medical staff is able to change the parameters of the virtual game (based on system indications and staff perceptions) during the rehabilitation procedure to adapt exercises and features according to patient progress and needs. In this way, SeeMe generates a setup whereby patients and medical staff are working together while the patient is at the medical center.

**Figure 2:**
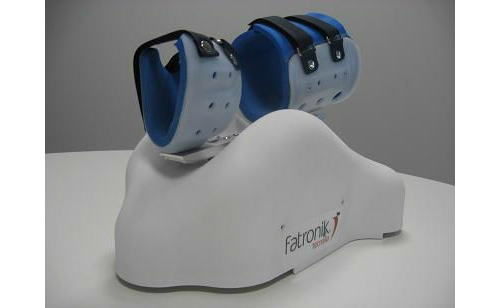
Arm Assist [15].

The last system we analyzed is ArmAssist [16], a functional prototype for at-home tele-rehabilitation of poststroke arm impairment. We aimed to provide a portable and easy-to-use device to enable effective rehabilitation (Figure 2). First, the clinical staff needs to decide when the patient is able to use the system independently at home. Then, the device is sent to the patient’s home and the rehabilitation process is monitored remotely with appropriate supervision and guidance. The device is integrated into a platform, named “TeleRehab” [17], which supports the phases of therapy planning, training, and assessment. The exercises to be completed with ArmAssist are divided into 2 types: assessment games and training games. The first type (1-2 minutes) is oriented toward working with an objective assessment of the range of movement, vertical force, and the ability to perform specific trajectories. The training games involve more complex activities (5-15 minutes), such as a word completion game. The results are stored and synchronized on a central server to be transmitted to the rehabilitation staff.

The studies mentioned above represent significant advances in rehabilitation processes through the use of new devices, game controllers, and movement-based interactions. However, the system presented in this paper offers new contributions that describe essential differences. In general, BDRehab is part of a complex system, named “Ubi4Health” [18], which provides a comprehensive solution for health care environments by operating from task management to rehabilitation process. Virtual Rehab is the closest work to the BDRehab system. The main difference is that Virtual Rehab uses games instead of real scenarios. Virtual Rehab embeds users in virtual games that help to improve their disabilities. In contrast, the BDRehab system involves patients in the real situation of a common daily activity, such as walking straight. In turn, WiiHab offers a parallel process designed to guarantee a rehabilitation program, but, similarly to Virtual Rehab, it involves patients in different games without engaging the patient in real daily activities. SeeMe represents a highly valid proposal to complete required rehabilitation programs at health care centers. In contrast, BDRehab is able to take the process to the home, which is where patients conduct activities of daily living. Therefore, patients can improve their condition without moving from home and without modifying their habits and comforts. Additionally, SeeMe requires the continuous presence of medical staff during the rehabilitation procedure, whereas BDRehab does not. BDRehab can be considered as an auxiliary application used at home. However, it must always be conducted under the adequate supervision of medical staff who manage the exercises to be completed and analyze the results, modifying parameters if necessary, but without the need to be physically present with the patient. ArmAssist represents a system that involves the use of a device worn by patients on their arms while performing the rehabilitation tasks. In contrast, patients are not required to wear any special equipment while performing their exercises with the BDRehab system. They interact with their own body with the use of Kinect, which captures their movements and postures. Therefore, BDRehab offers comfortable rehabilitation without the need to wear devices.

## Methods

### Balance Disorder Rehabilitation System

Considering how new technologies, and, more specifically, new interaction modes, can improve the conditions of patients in rehabilitation processes, we have developed a system focused on a specific functional exercise for persons with neurodegenerative diseases. The exercise consists of helping patients to recover from balance disorders by training them to walk straight. Consequently, the system is focused on a very specific objective. Patients are able to complete their therapy at home by performing the exercises defined by the medical staff without having to attend specialized centers. The main achievement is the incorporation of the therapy into the patients’ activities of daily living. Over time, the system will become an element of the patient’s home, and rehabilitation will be just another daily activity. However, BDRehab in no way replaces the medical staff responsible for deciding with whom and how each patient has to use the system to perform the rehabilitation tasks. Thus, the decision of when the patient is ready to carry out the process without further supervision is made by the medical staff.

BDRehab can be extended to an important variety of exercises in spite of being focused on just 1 specific exercise. Specifically, the system has been developed to be applied in functional exercises that represent daily life situations. The features of BDRehab support rehabilitation exercises based on physical movements. At present, the movements need to be wide and noticeable, owing to the limitations of the hardware used. However, the evolution in hardware technology has led to increasingly more sensitive devices, meaning that such intense movements will no longer be required for accurate detection.

Based on the information obtained in interviews and meetings with specialists in the field of physiotherapy, one of the keys to success in the recovery of patients with balance disorder, along with many other common problems, is the continuous repetition of the related rehabilitation exercise. This is a major challenge for the patient. These exercises appear simple, but actually entail great complexity. In most cases, the problem stems from the fact that, as one example, the patient is not aware that of losing balance on one side of the body while walking straight. The proposed system monitors patients in real time by allowing them to see themselves in a projected image that provides constant help for performing the series of exercises defined by the medical staff.

**Figure 3:**
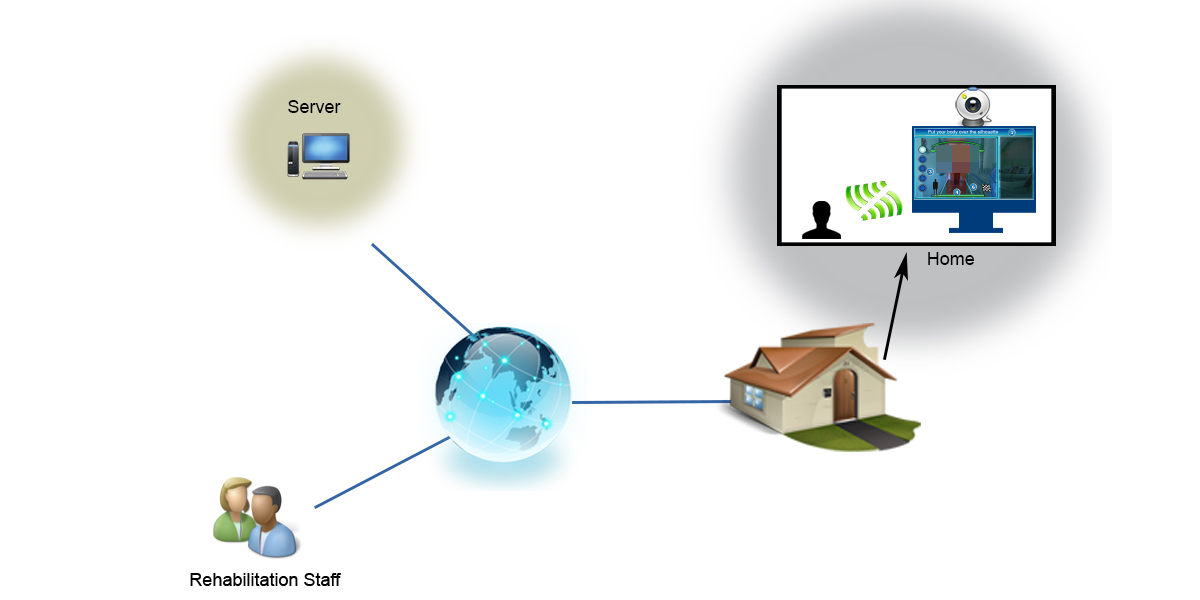
System Deployment.

The system is a well-defined deployment (see Figure 3) providing the new rehabilitation conditions. In particular, the system deployment is based on 2 connected points: the patient’s home computer and the system server. The first point represents the place where each Kinect device operates. To set up the system at the patient’s home, only a personal computer connected to a Kinect device and to a screen is needed. The personal computer requires a Web connection with a minimum bandwidth of 64 kbps to access the server, Windows 7 as the operating system, a dual core processor, and 2 GB of random access memory. In terms of visualization, the screen shows the system interface, which is the part of the system responsible for guiding the patient during the rehabilitation process. The server supplies and receives information to and from the software system running in the personal computer. Specifically, the information received is the usage statistics for each performed exercise to help medical staff analyze the evolution of the rehabilitation processes: attempts, errors, and the time needed to perform each session. The information supplied serves the purposes of parameter setting to customize the exercises for the patient.

The next subsections describe 3 essential elements of the system: (1) the exercises performed by the patients; (2) the system functionality with description of the fundamentals of the development and how it works; and (3) the interface that is needed to offer a system that helps patients and physiotherapists in the best possible way.

### Balance Exercises

A number of real rehabilitation exercises comprise the exercise to be performed by the patient. These are then checked by the system as an adaptation, including the most important aspects. The exercises to be conducted using the system were selected according to recommendations in the specialized literature [19,20] as well as the knowledge and experience of the physiotherapists involved. The experience of these professionals allowed the exercises to be adapted to the needs of rehabilitation patients.

One of the exercises consists of walking along a straight line without leaning sideways and going backward in the same way. In general, guide marks help the patients line up the area within which they have to walk. These marks are an essential element in reporting a deviation of which the patient is not aware.

The other exercise consists of improving balance through ensuring an upright posture of for a number of seconds without any swinging movements. In this case, the patient has to be in a specific posture: standing up, arms close to the body without lifting, back straight, and feet aligned with the shoulders.

The BDRehab system combines the previous exercises to generate a more complex exercise based on the first one. However, it monitors patients to ensure they maintain an upright posture while walking (as required by the second exercise) and also when still; in other words, when they start and finish the exercise.

**Figure 4:**
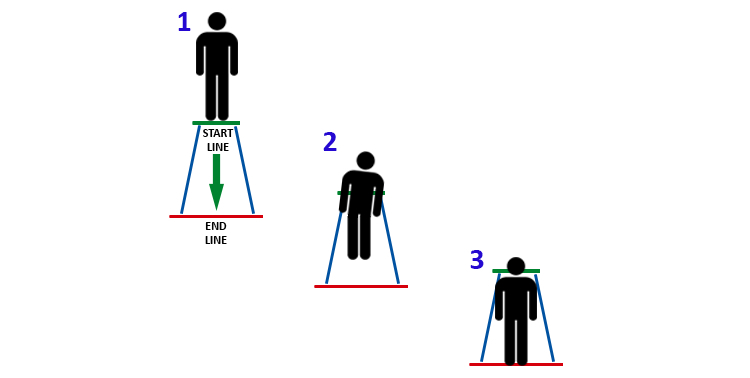
Steps to be performed during the BDRehab exercise: (1) to start in an upright posture; (2) to go along five-meter long straight line; and (3) to stop at the finish line and maintaining the upright posture.

Figure 4 shows the steps that comprise the complete BDRehab rehabilitation exercise. First, the patient has to stay still in an upright posture. Second, the patient has to walk along a 5-meter straight line without losing balance or deviating from the line. The last step consists of stopping at the finish line and maintaining an upright posture as at the start.

### System Functionality

The objective of the system is to check the posture of patients uninterruptedly during the exercise to guide them to perform the exercise described in the previous subsection. The system considers the exercise in 3 separate parts that involve different checks related to the postures and movements of the patient. To this end, the system maintains a variable named “step” to know which part of the exercise is performed and then the conditions to check. The system checks if the patient is at the initial position when the variable has the value 1. For value 2, the system checks if the patient is in the middle of the circuit and is balancing the body. For value 3, the system checks the patient’s final position and posture. In this way, the value of the variable increases from 1 to 3 after each step is checked. This monitoring process is carried out for each repeated exercise.

The monitoring process, which is recorded within a specific framework, comprises 2 parts. First, the system has to identify the posture of the patient. Posture identification is carried out by using the software development kit (SDK) provided by Microsoft [21]. This process is a key element, so an explanation of how the system performs the posture identification is essential. The SDK recognizes all 20 joints of the body that Kinect is able to identify. These points are supplied to the system through a code element that represents the skeleton and provides access to them through a set of 3 values referring to the position of the point in space (*x*-, *y*-, and *z*-axes). Once the system obtains this code element, the next step is to check if the patient is in the required posture, depending on the step of the exercise. To this end, the system considers that there are pairs of body parts that may be aligned with the vertical-horizontal coordinates. For example, the points related to the shoulders have to be at the same height during the exercise; otherwise, the patient is inclined to the side related to the shoulder that is in a lower position. However, the comparison allows a low error rate, which is checked through the difference of such coordinates within an error range.

**Figure 5:**
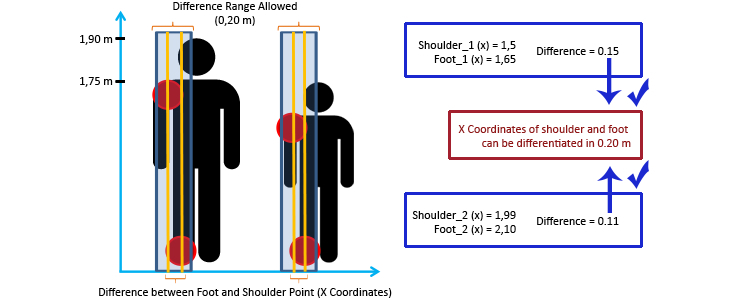
Example of the algorithm to check correct postures.

An essential feature of this framework is that it works independently of patient height. The main reason for this is that the comparison of coordinates is relative to each patient. The framework checks if a pair of body points is aligned in the *x*- or *y*-axis, which means the coordinates are the same in one of these axes or inside the error range allowed. In other words, the difference between the *x*/*y*-coordinates of 2 points, which may be aligned in the *x*/*y*-axis, must be zero or inside the error range, regardless of the height of the patient. Figure 5 shows an example in which the right feet and shoulders of 2 patients of different height are analyzed to establish if these body parts with the same *x*-coordinate are within an error range. The figure contains 2 marks for each patient to check if the posture is correct: (1) the range within which the difference of the *x*-coordinates of the related body points should be and (2) the difference itself. In this case, as the difference is within the range, the posture is correct for the 2 patients. The figure shows 2 patients for whom the framework has analyzed a specific posture, regardless of the patients’ difference in height. The important idea is that the 2 analyzed points should be of a similar value along the *x*-axis.

The use of difference of coordinates allows the framework to be independent of patient height. This feature avoids the need to calibrate the system for each user, a task that previously had to be carried out by every patient before starting the rehabilitation process. Therefore, patients are able to perform the rehabilitation session without any other complications.

The accuracy of the monitoring process is controlled through the management of a set of thresholds. The described framework and the whole system work under the control of each threshold, which can be customized by the physiotherapists. Increasing or decreasing each threshold makes the framework more or less strict. Specifically, the modification of the thresholds involves 2 factors: consideration of (1) wider or narrower error ranges as well as (2) the whole body of the patient or just some specific parts of it when checking the posture. This method modifies the precision of the system to detect postures and, consequently, the precision with which the patients have to perform the rehabilitation session. Therefore, the patients’ performance evolves favorably from the beginning to the end of the process. Initially, when patients find the exercise and the interaction more difficult, parameters are set at the minimum threshold; as they learn and overcome their limitations, the thresholds can be modified and increased accordingly. In this way, the thresholds are used to modify BDRehab based on the limitations on the patient’s movements. In technical terms, Kinect provides a degree of accuracy that is considered to be sufficient, natural, and adequate for the rehabilitation process carried out with the BDRehab system.

### System Interface

#### Overview

A good user interface is an essential element for patient acceptance of the system. Consequently, a rehabilitation system needs to be easy to understand and needs to guide patients adequately during the exercise under the physiotherapist’s supervision. As a result, patients will be more likely to include the system as a rehabilitation tool in their activities of daily living. In this sense, the system can guide each patient step by step, just as physiotherapists would do. Additionally, the interface can incorporate enough information to complete the specific exercise and also to help guide and correct users at any point of the process, giving them the corresponding feedback.

The system interface is based on the interaction provided by Kinect. The interface follows the generic usability guidelines for any application [22], as well as those defined by the Kinect device [23]. The generic guidelines refer to the visual aspect of the system, which must allow the patient to avoid uncomfortable working positions. Furthermore, the Kinect guidelines point the way to creating helpful interfaces controlled by natural movements, such as hand and head movements.

The type of user is a key component in analyzing the needs and appearance of the interface. Some patients may understand current technology, whereas others may know nothing about it. In this sense, analysis has shown that it is fundamental that the interface have a well-defined set of warnings, notifications, and signals. At the same time, the system can show the information on the screen together with appropriate metaphors to complete the indications. Therefore, the system has to constantly let patients know exactly what to do and how to do it, particularly because the process of the exercise is controlled by patient interaction.

**Figure 6:**
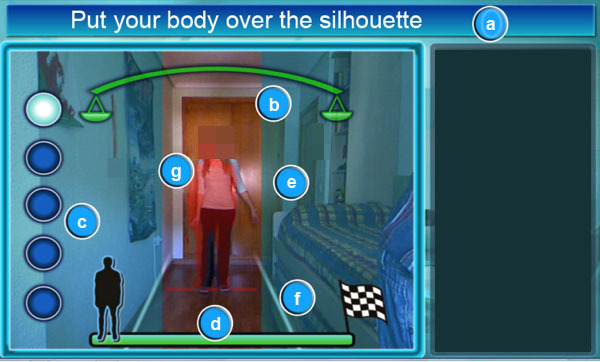
System screenshot which shows user instructions in order to complete the rehabilitation exercise. The interface elements in the screenshot are: (a) current task, (b) balance level, (c) attempts, (d) level of completion, (e) margins of movement, (f) walking lines and (g) patient's silhouette.

**Figure 7:**
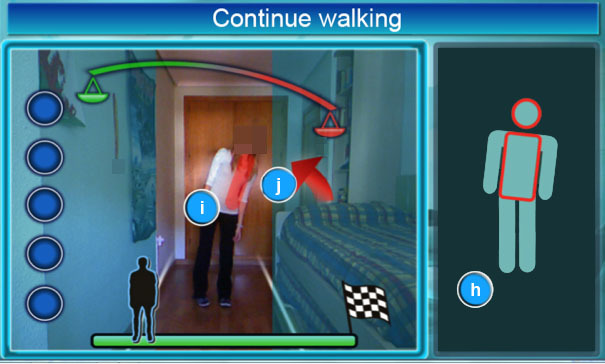
System screenshot which shows an overview of a complete rehabilitation exercise. The interface elements in the screenshot are: (h) animation area, (i) misaligned bones corrector and (j) corrective arrows.

Figures 6 and 7 show screenshots of the system in which it is possible to observe the information given to patients during the rehabilitation process. Each element of the interface is outlined in the paragraphs below.

#### Current Task of the Exercise to be Performed

The patient needs to know what to do at all times. The system analyzes the development of the exercise by studying the distance covered by the patient. First, the system checks that the user is at the starting point. If the patient is not at the required point, the system indicates that the patient needs to be there. Then, the system indicates that the next step is to walk until the end of the rehabilitation route. Finally, the system determines the end of the exercise when the patient reaches the end of the virtual line that has been followed.

#### Balance Level

This mark is a key piece of information. A set of scales that inclines to the side on which the patient is leaning while walking shows the patient’s balance. This is an effective way of showing the correctness of the position. If the user inclines the body excessively, the balance is inclined and the color of the affected side changes to red.

#### Attempts

The system records the attempts the user has made to complete the exercise, based on the physiotherapist’s indications.

#### Level of Completion of the Complete Exercise

Patients need to know which stage of the exercise they are at. This signal marks a man in a line showing the progress made. At the left is the starting point, and at the right is the finishing point.

#### Margins of Movement

These elements are 2 squares that diffuse the image to limit the action range of the patient during the walk. The margins widen as the patient nears the finishing point to generate a correct sense of depth. If the patient touches any margin, it will light up in red to indicate what is happening.

#### Walking Lines

Walking elements create margins to show the walking space available to perform the exercise. If the user touches any of the lines, the system lights up the corresponding margin in red to show what is happening.

#### Patient’s Silhouette

This element helps patients locate their bodies when adopting the initial posture. Specifically, the silhouette uses a section in red to represent the possible position of the patient.

#### Animation Area

This area contains the animations with a guide highlighted in red. The animation shows the patient the next action to be performed. This area includes information regarding the postures and movements that need to be corrected.

#### Misaligned Bones Corrector

This element indicates badly positioned joints during the current exercise using a semitransparent red surface. In this way, the corrector helps the patient understand the errors they make.

#### Corrective Arrows

The corrective arrows indicate the side toward which the patient’s posture must be corrected during the exercise. Figure 7 shows how posture must be corrected to the appropriate side.

#### Conclusions

This information appears when the patient finishes each attempt. This element gives a list of what needs to be corrected in the next step. If the attempt is performed correctly, the system “congratulates” the patient.

It is important to highlight that, to avoid causing the patient unnecessary stress, the time recording is not shown to the patient. However, BDRehab internally collects the duration of each exercise attempt, which is seen only by the medical staff. They can use the time recording as additional information to modify the accuracy of the monitoring process through the set of thresholds and also to better assess the evolution of the patients.

The possibility of using the patient’s voice to control the functionality of the system completes the interface. This capacity is provided by the Kinect device, which can interact with users through their voice by offering a microphone component and related software with voice recognition capability. This creates a better environment for users with arm mobility problems, in which they can operate the interface using oral instructions. Primarily, it allows users to manage the system by using preferences or simple actions such as indicating that they are ready to start.

## Results

The evaluation of the system has been performed following a user-centered approach. The process comprised 2 iterations. The outcomes of both evaluations were utilized to develop the system. Taking this into account, the version of the system described in this paper reflects most of the improvements that the evaluation process provided as an iterative process.

Five simulated patients took part in each iteration of the evaluation process. According to Nielsen and Landauer [24], 5 participants are sufficient to find 85% of errors and/or problems. The group comprised 2 teenagers (14 and 16 years old), 1 adult (31 years old), and 2 older adults (70 and 75 years old). The environment consisted of a 7×4-meter room in which a 24-inch screen connected to a Kinect device was installed at a height of 1 meter.

Two therapists were present during the whole evaluation process. They were responsible for ensuring that the process was carried out correctly. The active involvement of the therapists was a key factor in the success of the evaluations, because, unlike the present authors, they are experts in balance disorder rehabilitation and were better able to manage the process. The main objective was to know the effectiveness of the system. Therapists are fully familiar with the behavior patterns and limitations of the patients and were thus able to guide the participants to imitate the movements of a real patient with great precision.

The evaluation was recorded using a video camera to collect each participant’s reaction when using the system. In addition, the image displayed on the screen was recorded to show the relationship between the participant’s reactions and what the system was displaying on the screen.

The evaluations were performed in 2 iterations. The first one was performed without any prior explanation to the patients about the operation mode of the BDRehab system. In this way, we aimed to obtain information about the intuitiveness of the interface elements. The participants had to detect, identify, and interpret each element and functionality to complete one attempt of the rehabilitation exercise. Next, a complete explanation about BDRehab was given to the participants, and they then attempted the exercise 4 times. Finally, the participants were asked about the intuitiveness and simplicity of the system and what they thought could be eliminated.

**Figure 8:**
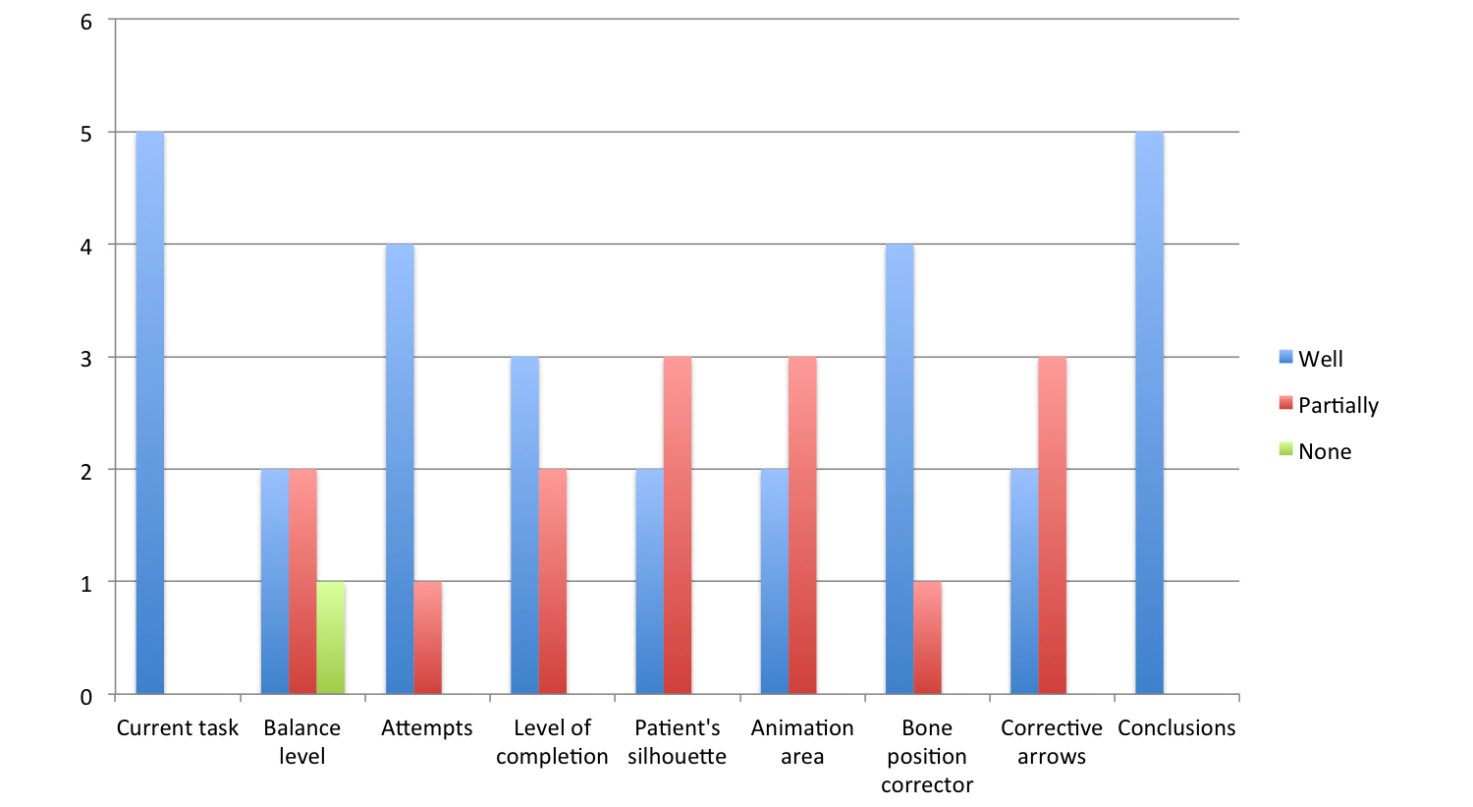
Identification of the interface elements.

The outcomes provided interesting feedback that provided a basis on which to make improvements to the system following the established user-centered design approach. We collected participants’ impressions, which are graphically depicted in Figure 8. In general, most participants found the system easy to work with. There was only one case in which a participant could not identify an element of the interface, which was the balance level. All other participants were able to identify the elements totally or partially. However, some initial interface elements (44%, 4 of 9) presented difficulties, because the partial identification was greater than or equal to the total identification. Specifically, 4 elements of the interface were involved in most problems: balance level, animation area, patient silhouette, and corrective arrows.

Only one color was used for the balance level, and many participants felt the color should change when their balance moved toward one side, indicating a motion error. The animation area confused older people when the examples showed an image in a sideways position. The same participants had problems following the patient silhouette because some points vibrated, causing them to be easily distracted. According to these findings, we and the therapists improved the following 4 aspects of the system. (1) The balance level was changed to green if the posture is correct and red if the posture moves to an incorrect side. (2) The animation area was changed, using new animations that better represented the postures to be repeated. (3) The code of the system was analyzed and corrected to avoid any vibration during the exercises. (4) The corrective arrows were modified to make them curved to represent the curvature of the back.

During the first iteration of the evaluation, the 2 therapists conducted a thorough analysis of the system. As a result, they considered the system a useful tool for the patients during their rehabilitation programs. The system avoids the need for patients to frequently attend medical centers and allows them to complete rehabilitation at home if their conditions are suitable. Despite positive impressions, the therapists suggested a set of improvements to be made, taking into account how patients usually work. First, they found it necessary to consider the possibility of working with patients with disabilities in other parts of the body. Specifically, the system was highly restrictive and failed if patients did not move an arm during the exercise. The solution found was to allow therapists to customize the body parts to be checked during the exercise. In addition, the therapists emphasized the need to improve the method of indicating the direction of the user during the exercise. The main reason was that many patients have difficulty in maintaining the same direction from the starting point to the end. Hence, we included 2 elements in the interface: the margins of movements and the walking lines (Figure 6). An additional consideration was the need to include more information about the exercise based on errors made. In this way, a more detailed report about the errors made during the exercise was included in the conclusions element.

The second iteration consisted of using a new version of the BDRehab system with many differences from the initial one. The objective was to know whether the redesigned interface provided a suitable set of elements and consequently whether the results of the first iteration had been interpreted correctly. The same participants attempted and successfully completed the rehabilitation exercise 4 times. Two additional improvements were also established. The older participants required audio instructions, as they experienced problems when listening to the instructions while checking their own exercises on the screen. This modification was introduced as an essential element because it includes additional information that helps patients complete the rehabilitation exercise without cluttering the user interface. The therapists found the system appropriate for application in a rehabilitation program but considered that some patients prefer not to see their own body displayed on the screen, so they thought the system would benefit from a virtual environment to eliminate real images. This concern is an interesting possibility for future research.

**Figure 9:**
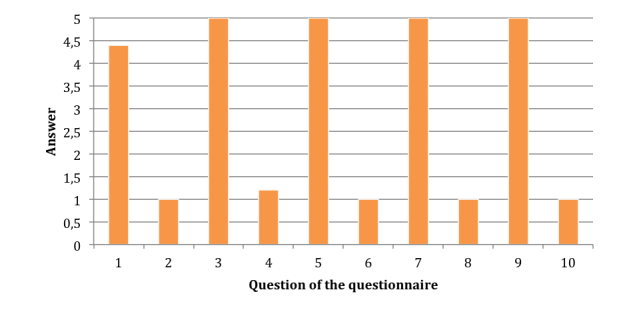
System Usability Scale (SUS) Results.

Finally, we tested the usability of the latest version of the system, in terms of user satisfaction, by using the System Usability Scale (SUS) [25]. The results of this test are shown in Figure 9. SUS scores have a range of 0 to 100, and we achieved a score of 98, which means that the usability of the system is reasonably high.

## Discussion

In this paper, we present a system designed to improve rehabilitation conditions of people with balance disorders. In particular, the system serves as a rehabilitation tool for patients who need training to walk straight. The main contribution comes from the fact that the system allows patients to perform the rehabilitation process at home under the supervision of physiotherapists.

The proposal presents significant benefits from two points of view. The first is that it takes into account the perspective of the medical staff, who find the system to be a useful time-saving tool. The time gained increases the capacity of medical staff to attend to patients more intensively, defining specific parameters for the exercise for each patient. The system partially replaces the presence of therapists during the performance of the exercise. Therapists are then able to focus on the results and the way to improve each rehabilitation process. The rehabilitation exercise is usually highly repetitive. This means that the constant presence of the physiotherapist is not required, because instructions are provided by the system. These instructions provide patients with enough information for them to know what they are doing incorrectly and how to correct the action. In this way, the system contains different ways to alert the patients without overwhelming them. The easiest instructions, and generally the most common ones, appear on the screen, adding color to the part of the exercise that is being wrongly completed. For example, the system uses an arrow to show the way to correct balance (see Figure 7). Other indications are more intrusive if the problem to be corrected could affect the patient’s health. For example, patients may fall if they do not correct a certain action. Although rehabilitation requires constant monitoring, traditionally with the physical presence of a therapist, this system constantly monitors the repetitive exercises, replicating the full-time attention usually provided by a therapist.

On the one hand, one might think that, without the presence of medical staff patients miss out, to some degree, on the personal treatment that can often be of great comfort. On the other hand, the patients are able to make progress at home with their family or people close to them. Despite the fact that medical staff is not physically present, they receive updated information about the progress of each patient. BDRehab collects information about patients’ progress and generates statistical data on how patients are performing in each attempt during the rehabilitation process, allowing patients to control their progress and analyze the results in depth. Regarding how medical staff can assess the progress of the rehabilitation sessions, the system stores the data produced during the rehabilitation session at the patient’s home. The therapists can then analyze these data and modify the parameters of the exercises accordingly to adapt the exercises to the progress of the patient.

The second point of view to be considered is that of the patient. The system avoids the need for patients to attend medical or specialty centers for rehabilitation. In this way, the patients can remain at home, a familiar and more comfortable environment, which is certainly of physiological benefit. Additionally, patients can conduct the rehabilitation process in an independent and less invasive way without the need to depend on others more than is strictly necessary. Therefore, they can organize their progress at their own leisure, based on the steps marked out by medical staff. These 2 benefits represent a significant development in rehabilitation, especially taking into account that some patients have to continue the rehabilitation process for the rest of their lives.

This proposal builds upon movement-based interaction through Kinect devices and a framework that analyzes patients’ movements. The framework studies how each patient performs the exercise and, depending on this performance, creates appropriate instructions to provide feedback to the patient to correct and guide them. The analysis consists of checking the posture and movements of the patient in the 3 steps into which the rehabilitation exercise is divided. The first and last steps help check if the patient has the correct posture at the beginning and end of the exercise: standing up, arms close to the body, back straight, and feet aligned with the shoulders. The intermediate steps consist of analyzing how the patient walks from the beginning to end. In this sense, the system searches for straight movements. Feedback instructions are fundamental to help the patient during the exercise. The data gathered during the performance of the exercise (eg, time, number of errors) are stored so the therapist can supervise the evolution of the patient’s progress and adapt the exercises accordingly.

The system has been evaluated by 2 therapists with outstanding experience in balance disorder rehabilitation and by 5 people representing real patients. The outcomes of the evaluation were positive, indicating that the system could be applied in a real rehabilitation program as a useful aid for patients to follow the therapy at home and also as a suitable auxiliary tool for therapists to supervise the evolution of patients’ progress without the need to be physically present all the time. Furthermore, the therapists involved in the research provided us with interesting ideas for improving the system, which were crucial to achieving the final version described in this paper.

## Abbreviations

SDK: software development kit
SUS: System Usability Scale
